# On Infant Feeding and Infant Foods

**Published:** 1888-12

**Authors:** 


*On Infant Feeding and Infant Foods.* By JAMES MCNAUGHT, M.D. (Edin.), M.R.C.S. (Eng.) London : JOHN HEYWOOD. 1888.

The few pages forming this booklet are a handy compendium of the well-known facts concerning the subjects therein treated.

In hand-feeding the author seems to recognise only the convenient, but dangerous, bottle with long tube, which has to be cleaned by brush. The earlier form of bottle with teat attached direct is much safer, but, of course, involves more trouble.

The chapter on Artificial Feeding contains much useful information about its latest developments. Attention is directed to the early " Symtoms *(sic)* of Rickets," and the connection of this condition with improper feeding. It would be an addition to the terrors of maternity and a needless worry to the mother to follow the author's direction, that after the first three days "the child should be applied to the breast every two hours, night and day, for the first month." (p. 36).

This tiny volume affords one more instance of the bad taste of overloading a title-page with unrequired information about the author; but altogether it is characterised by much common-sense, and its facts are not obscured by unnecessary verbiage.

